# Dietary pattern adherence in association with changes in body composition and adiposity measurements in the UK Biobank study

**DOI:** 10.1016/j.orcp.2023.05.008

**Published:** 2023

**Authors:** Anna K. Sweetman, Jennifer Carter, Aurora Perez-Cornago, Min Gao, Susan A. Jebb, Carmen Piernas

**Affiliations:** a Nuffield Department of Population Health, University of Oxford, Oxford, UK; b NIHR Oxford Biomedical Research Centre, University of Oxford, Oxford, UK; c Cancer Epidemiology Unit, Nuffield Department of Population Health, University of Oxford, Oxford, UK; d Nuffield Department of Primary Care Health Sciences, University of Oxford, Radcliffe Primary Care Building, Radcliffe Observatory Quarter, Woodstock Road, Oxford OX2 6GG, UK; e Department of Biochemistry and Molecular Biology II, Faculty of Pharmacy, Center for Biomedical Research, University of Granada, Granada, Spain

**Keywords:** Reduced rank regression, Fat mass, Skeletal muscle mass, BMI, Waist circumference, Cohort study

## Abstract

**Background:**

Unhealthy dietary patterns (DP) have been frequently linked to avoidable ill-health, mediated in part through higher body mass index. However it is unclear how these patterns relate to specific components of body composition or fat distribution, and whether this may explain reported gender differences in the relationship between diet and health.

**Methods:**

Data from 101,046 UK Biobank participants with baseline bioimpedance analysis and anthropometric measures and dietary information on two or more occasions were used, of which 21,387 participants had repeated measures at follow up. Multivariable linear regressions estimated the associations between DP adherence (categorised in quintiles Q1–Q5) and body composition measures adjusted for a range of demographic and lifestyle confounders.

**Results:**

After 8.1 years of follow-up, individuals with high adherence (Q5) to the DP showed significantly positive changes in fat mass (mean, 95 % CI): 1.26 (1.12–1.39) kg in men, 1.11 (0.88–1.35) kg in women vs low adherence (Q1) − 0.09 (− 0.28 to 0.10) kg in men and − 0.26 (− 0.42 to − 0.11) kg in women; as well as in waist circumference (Q5): 0.93 (0.63–1.22) cm in men and 1.94 (1.63, 2.25) cm in women vs Q1 − 1.06 (− 1.34 to − 0.78) cm in men and 0.27 (− 0.02 to 0.57) cm in women.

**Conclusion:**

Adherence to an unhealthy DP is positively associated with increased adiposity, especially in the abdominal region, which may help explain the observed associations with adverse health outcomes.

## Introduction

Unhealthy dietary patterns have been linked to a higher risk of morbidity and premature mortality [1], [2], [3]. Among British adults, a dietary pattern (DP) derived through reduced-rank regression in the UK Biobank study explained maximum variability in a set of response variables (dietary energy density, saturated fat, free sugars and fibre intake) and was characterised by high intakes of chocolate confectionery, butter and refined carbohydrates, and low intakes of fruits and vegetables [2]. This DP showed significant positive associations with major health outcomes in prospective analyses, including cardiovascular disease (CVD), type 2 diabetes and all-cause mortality [2], [3]. However, it is not known whether this dietary pattern is mediated through specific effects on body composition, including fat mass (FM) and skeletal muscle mass (SMM), or in general adiposity and its distribution, namely body mass index (BMI), waist circumference (WC) and waist-to-hip ratio (WHR).

Our review of the literature shows that the evidence for an association between dietary patterns and body composition or adiposity measures in adults is limited to small-scale studies, the majority of which are cross-sectional and none were conducted in the UK adult population (Supplementary Table 1). Three small studies (n < 1080) in populations from the US and China have assessed prospective associations between dietary patterns and changes in body composition, generally suggesting that adherence to “western” or “modern” dietary patterns are associated with significant increments in adiposity [4], [5], [6]. In addition, none of these previous studies has derived DPs through reduced rank regression (RRR), which is an exploratory approach with the advantage of using a priori knowledge of nutrient-disease associations to derive data-driven DPs [7].

Using data from the UK Biobank study, we aimed to investigate prospective associations between adherence to an unhealthy dietary pattern and changes in body composition measures (FM and aSMM), as well as classical measures of adiposity and its distribution (BMI, WC and WHR). Secondly, we investigated associations with baseline measures of body composition and effect modification by sex, since differences in body composition and adiposity may help to explain observed differences in the risk of some non-communicable diseases between men and women.

## Materials and methods

### Study design

The UK Biobank is a large population-based prospective cohort study, designed to provide an extensive breadth of data on genetic and non-genetic factors, to allow for research into their association with disease [8], [9]. Between 2006 and 2010, invitations to participate in the study were mailed to individuals aged 40–69 living within 25 miles of the 22 assessment centres (n = 9.2 million). 5.5 % of invitees responded and 502,664 participants were recruited. Participants attended an assessment clinic, where they completed a touch-screen questionnaire and had a face-to-face interview with a study nurse, which covered sociodemographic characteristics, family and personal history of illness, early life exposures, psychosocial factors, environmental factors, lifestyle, health status and cognitive function. Participants also had physical measurements taken (height (cm), weight (kg), bioimpedance analysis (BIA), hip and waist circumference (cm)) and had biological samples collected (blood, urine and saliva) [9].

### Dietary intake and exposure definition

The Oxford WebQ, a 24-h online dietary assessment tool, was completed at baseline and up to four occasions at follow-up) by a subsample of individuals who provided an email address at recruitment [10]. It has been validated against biomarkers [11] and compared to interviewer-administered 24 h recalls [12] and showed acceptable reproducibility when using at least two dietary assessments [13]. To better reflect usual intakes, only participants who had completed a dietary assessment on two or more occasions were included in the present analyses. Also, individuals with implausible values of total energy intake were excluded; this was calculated by using the ratio of reported energy intake (EI) to estimated energy requirement (EER) calculated as basal metabolic rate [14], i.e. the EI:EER ratio [15]. A 95 % Confidence Interval (CI) for the accuracy of EI:EER ratio was calculated by taking into account the amount of variation inherent in the methods used to estimate EI and EER [16]. Individuals were classified as plausible reporters (EI:EER within the 95 % CI), under-reporters (EI:EER < lower limit of 95 % CI EI:EER) or over-reporters (EI:EER > upper limit of 95 %CI EI:EER).

Food and drinks recorded in the WebQ were classified into 50 food groups, based on their nutrient profile or culinary use [17]. Daily nutrient intakes were then calculated for each individual using the UK Nutrient Databank (2013) [18]. The dietary pattern (DP) used as the exposure in these analyses was previously derived in the UK Biobank population using reduced rank regression [2]. This DP explains the maximum variation (43 %) in several response variables (energy density (kJ/g), saturated fat (% total energy), free sugar (% total energy), and fibre density (g/MJ)), chosen because there is evidence that they play a role in the development of CVD and mortality. This DP was characterised by high intakes of chocolate confectionery, butter and refined carbohydrates, and low intakes of fruits and vegetables. Each participants’ average intake of different food groups was then calculated, and respondents were assigned a z-score using a weighted combination of their standardised food group intakes. A higher intake of food groups having a positive factor loading increases the dietary pattern z-score, while a higher intake of food groups with negative factor loadings decreases the dietary pattern z-score. The higher the z-score, the stronger the adherence to the DP (Supplementary Fig. 1**)**. This z-score was categorised into quintiles and used as the exposure of interest.

### Body composition and adiposity measures

At the baseline assessment centre visit, bioimpedance analysis (BIA) was performed using a Tanita BC418MA body-composition analyser (Tanita, Tokyo, Japan). BIA provides measurements which are specific to regions of the body (trunk, arms, or legs). Body composition measures used in these analyses included the appendicular skeletal muscle mass (aSMM; the skeletal muscle mass in the four limbs) and FM (derived from BIA for the whole body (trunk, arms, and legs)). Our rationale for using aSMM as opposed to fat-free mass, or whole-body muscle mass is because aSMM is less likely to be confounded by FM. For example, abdominal FM leads to more skeletal muscle mass in the trunk (for structural support) thus excluding skeletal muscle mass in the trunk will reduce the amount of confounding in the model [19]. We also excluded participants of a non-white ethnicity because BIA estimates are derived from algorithms coming from predominantly white populations, and it is not recommended to use prediction equations that have not been validated for other ethnicities [20].

Other adiposity measures included in these analyses were the body mass index (BMI), waist circumference (WC) and the waist to hip ratio (WHR). BMI was calculated by dividing the weight (kg) by the height squared (metres). Height was measured using the Seca 202 device (in a barefoot, standing position). Waist and hip circumference (cm) were measured using the Wessex non-stretchable sprung tape measure. WHR ratio was calculated by dividing waist circumference by hip circumference.

Different subsamples of the baseline population returned at 3 different time points for follow up measurements (2012–2013; 2014 and 2019 [21]). Repeated body composition measures were taken using the same methods described above.

### Statistical analysis

We used multivariable linear regression models to estimate predicted marginal means for the associations of adherence to the DP with prospective changes in FM, aSMM, BMI, WC and WHR, between baseline and follow up; as well as cross-sectional analyses using baseline measures of FM, aSMM and BMI, WC and WHR. The cross-sectional mean estimates of FM, aSMM and BMI were logarithmically transformed to satisfy model assumptions and normalise distributions and the β coefficients were exponentiated to yield geometric mean estimates of these variables in each DP quintile and corresponding 95 % confidence intervals (CIs). Arithmetic mean estimates were presented for all other outcomes. Adjustments were made for age (5-year age groups), height (cm), Townsend deprivation index (quintiles), education (‘higher degree’ (college or university degree, or professional qualifications), ‘any school degree’ (A levels, AS levels, O levels, GCSEs or CSEs), ‘vocational qualifications’ (NVQ, HND or HNC), or ‘none of the above’), physical activity (‘High’ defined as ≥ 3000 MET-min/week or vigorous activity on ≥ 3 days; ‘moderate’ defined as ≥ 600 MET-min/week or vigorous activity on ≥ 20 min/day; ‘low’ defined as < 600 MET-min/week), smoking status (current, previous, never), prior cancer (> 5 years ago), and menopausal status in females. For models predicting aSMM and FM, we mutually adjusted for FM in the aSMM models, and vice versa. For models predicting changes between baseline and follow up, the baseline value of each exposure of interest was also adjusted.

To determine whether there was heterogeneity by sex in these associations, an interaction term between sex and DP quintile was added to the final models, and likelihood ratio tests (assessing the goodness of fit of models with vs without the interaction term) were performed to assess evidence of effect modification. Significant interactions by sex were identified *(P <* 0.05) for several of the associations, and thus stratified results were presented for all outcomes.

We conducted three sensitivity analyses. Firstly, since BIA may not be accurate for people with a high BMI [22], we repeated the main analyses after excluding 964 participants with severe obesity (≥ 40 kg/m^2^, n = 100,082). Secondly, an analysis was run which only included participants who had their first 24-h dietary assessments at baseline (plus at least another 24-h dietary assessment during the follow-up) at the same time as their Tanita measurements were taken (n = 26,751). A third sensitivity analysis included people with 3 or more 24-h dietary assessments (n = 32,512), to assess whether a more accurate measure of usual dietary intake (reduced random error) affected the findings. All statistical analyses were performed with the use of Stata release 16.1 (Stata-Corp, College Station, Texas, USA), and 2-sided *P* values < 0.05 were considered significant.

## Results

### Baseline characteristics

**Fig. 1:**
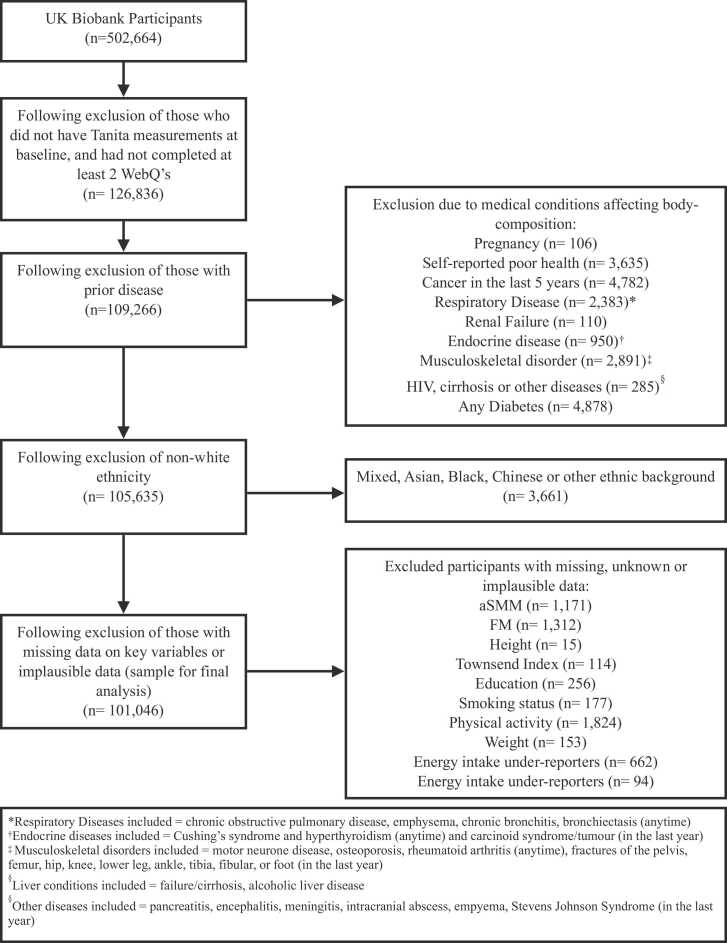
**Participant flow chart.** *FM – Fat Mass; aSMM – Appendicular Skeletal Muscle Mass.

A total of 126,836 participants had Tanita measurements at baseline and had completed at least 2 24-h dietary assessments (Fig. 1). From this, we excluded individuals with reported pregnancy or prior relevant conditions such as self-reported poor health, cancer in the last 5 years, respiratory disease, renal failure, endocrine diseases, musculoskeletal disorders, HIV, cirrhosis, other diseases and diabetes (N = 17,570), non-white ethnicity (N = 3661), missing data on exposure, outcome or key confounders (N = 5022) as well as those with implausible values of total energy intakes (N = 756). The sample size for final analysis was 101,046, of which n = 21,387 had repeated body composition measurements at follow up.

Table 1 (women) and Table 2 (men) show characteristics of the participants who have baseline body composition and adiposity measures across DP quintiles. The mean age at recruitment was 55.4 (SD 7.7) and 56.6 years (SD 7.9) for women and men respectively. For both sexes, in DP Quintile 5 (Q5) there was a greater proportion of never smokers, and a lower proportion of current smokers than in DP Quintile 1 (Q1). Furthermore, participants in Q1 were more likely to have higher education and less likely to be in the “low” physical activity group than those in Q5.

**Table 1:** Baseline characteristics of females (n = 56,391), overall and by DP quintiles.

		Dietary pattern quintile					
Characteristic	Total	Quintile 1	Quintile 2	Quintile 3	Quintile 4	Quintile 5	p-value
Number of Participants	N = 56,391	N = 11,279	N = 11,278	N = 11,278	N = 11,278	N = 11,278	
Age at recruitment (years), mean (SD)	55.35 (7.69)	56.92 (7.23)	56.51 (7.35)	55.71 (7.59)	54.68 (7.73)	52.93 (7.84)	< 0.001
Height (metres), mean (SD)	163.75 (6.11)	163.60 (6.07)	163.59 (6.08)	163.66 (6.07)	163.91 (6.11)	163.98 (6.23)	< 0.001
Dietary pattern z_score, mean (SD)	-0.33 (1.35)	-2.26 (0.83)	-0.96 (0.22)	-0.28 (0.18)	0.36 (0.20)	1.48 (0.66)	< 0.001
aSMM (kg), mean (SD)	18.30 (2.21)	18.19 (2.16)	18.11 (2.09)	18.23 (2.12)	18.35 (2.22)	18.64 (2.40)	< 0.001
FM (kg), mean (SD)	25.30 (9.28)	24.55 (9.15)	24.62 (8.66)	25.05 (8.92)	25.50 (9.22)	26.79 (10.22)	< 0.001
BMI (kg/m^2^), mean (SD)	26.04 (4.63)	25.73 (4.53)	25.69 (4.31)	25.92 (4.44)	26.11 (4.61)	26.78 (5.13)	< 0.001
WC (cm), mean (SD)	82.19 (11.45)	81.24 (11.26)	81.31 (10.86)	81.88 (11.05)	82.50 (11.41)	84.02 (12.37)	< 0.001
WHR, mean (SD)	0.80 (0.07)	0.80 (0.07)	0.80 (0.06)	0.80 (0.07)	0.81 (0.07)	0.81 (0.07)	< 0.001
Townsend Deprivation Index, n(%)	-1.71 (2.75)	-1.77 (2.73)	-1.81 (2.70)	-1.79 (2.68)	-1.73 (2.75)	-1.44 (2.86)	< 0.001
1 (least deprived)	11,259 (19.97 %)	2342 (20.76 %)	2332 (20.68 %)	2305 (20.44 %)	2284 (20.25 %)	1996 (17.70 %)	
2	11,399 (20.21 %)	2339 (20.74 %)	2355 (20.88 %)	2302 (20.41 %)	2292 (20.32 %)	2111 (18.72 %)	
3	11,600 (20.57 %)	2247 (19.92 %)	2327 (20.63 %)	2344 (20.78 %)	2355 (20.88 %)	2327 (20.63 %)	
4	11,460 (20.32 %)	2291 (20.31 %)	2251 (19.96 %)	2308 (20.46 %)	2235 (19.82 %)	2375 (21.06 %)	
5 (most deprived)	10,673 (18.93 %)	2060 (18.26 %)	2013 (17.85 %)	2019 (17.90 %)	2112 (18.73 %)	2469 (21.89 %)	
Education, n(%)							< 0.001
Higher degree	29,721 (52.71 %)	6526 (57.86 %)	6296 (55.83 %)	5991 (53.12 %)	5806 (51.48 %)	5102 (45.24 %)	
Any school degree	17,807 (31.58 %)	3188 (28.26 %)	3425 (30.37 %)	3584 (31.78 %)	3570 (31.65 %)	4040 (35.82 %)	
Vocational qualification	5613 (9.95 %)	996 (8.83 %)	933 (8.27 %)	1064 (9.43 %)	1236 (10.96 %)	1384 (12.27 %)	
None of the above	3250 (5.76 %)	569 (5.04 %)	624 (5.53 %)	639 (5.67 %)	666 (5.91 %)	752 (6.67 %)	
Smoking status, n(%)							< 0.001
Never	34,644 (61.44 %)	6989 (61.96 %)	7012 (62.17 %)	6983 (61.92 %)	6990 (61.98 %)	6670 (59.14 %)	
Previous	18,569 (32.93 %)	3883 (34.43 %)	3811 (33.79 %)	3746 (33.22 %)	3655 (32.41 %)	3474 (30.80 %)	
Current	3178 (5.64 %)	407 (3.61 %)	455 (4.03 %)	549 (4.87 %)	633 (5.61 %)	1134 (10.05 %)	
Physical activity, n(%)							< 0.001
Low	11,866 (21.04 %)	1732 (15.36 %)	2073 (18.38 %)	2396 (21.24 %)	2603 (23.08 %)	3062 (27.15 %)	
Moderate	30,290 (53.71 %)	6040 (53.55 %)	6284 (55.72 %)	6072 (53.84 %)	6118 (54.25 %)	5776 (51.21 %)	
High	14,235 (25.24 %)	3507 (31.09 %)	2921 (25.90 %)	2810 (24.92 %)	2557 (22.67 %)	2440 (21.64 %)	
Cancer history (yes), n(%)	3319 (5.89 %)	733 (6.50 %)	690 (6.12 %)	628 (5.57 %)	657 (5.83 %)	611 (5.42 %)	0.004
Menopause (yes), n(%)	32,725 (58.03 %)	7355 (65.21 %)	7181 (63.67 %)	6725 (59.63 %)	6256 (55.47 %)	5208 (46.18 %)	< 0.001

DP – Dietary Pattern; FM – Fat Mass; aSMM – Appendicular Skeletal Muscle Mass; BMI – Body Mass Index; WC – Waist Circumference; WHR – Waist-to-Hip Ratio. *Pearson’s chi-squared tests were used to compare the distribution between z-score for categorical variables, and ANOVA was used to compare the z-score for continuous variables.

**Table 2:** Baseline characteristics of males (n = 44,655), overall and by DP quintile.

Characteristic	Total	Quintile 1	Quintile 2	Quintile 3	Quintile 4	Quintile 5	p-value
Number of Participants	N = 44,655	N = 8931	N = 8931	N = 8931	N = 8931	N = 8931	
Age at recruitment (years), mean (SD)	56.58 (7.92)	58.11 (7.46)	57.74 (7.63)	56.88 (7.77)	55.98 (7.93)	54.22 (8.16)	< 0.001
Height (metres), mean (SD)	176.89 (6.62)	176.71 (6.56)	176.76 (6.61)	176.72 (6.57)	177.04 (6.57)	177.24 (6.75)	< 0.001
Dietary pattern z_score, mean (SD)	0.42 (1.49)	-1.64 (0.85)	-0.30 (0.23)	0.42 (0.20)	1.15 (0.24)	2.49 (0.82)	< 0.001
aSMM (kg), mean (SD)	27.00 (3.50)	26.60 (3.44)	26.69 (3.41)	26.91 (3.37)	27.17 (3.45)	27.61 (3.73)	< 0.001
FM (kg), mean (SD)	20.90 (7.42)	19.87 (7.28)	20.46 (7.19)	20.96 (7.11)	21.25 (7.30)	21.96 (7.98)	< 0.001
BMI (kg/m^2^), mean (SD)	27.00 (3.73)	26.53 (3.61)	26.73 (3.62)	27.01 (3.60)	27.16 (3.69)	27.56 (4.03)	< 0.001
WC (cm), mean (SD)	94.92 (10.34)	93.28 (10.22)	94.25 (10.12)	94.96 (10.03)	95.47 (10.19)	96.66 (10.79)	< 0.001
WHR, mean (SD)	0.92 (0.06)	0.91 (0.06)	0.92 (0.06)	0.92 (0.06)	0.93 (0.06)	0.93 (0.06)	< 0.001
Townsend Deprivation Index, n(%)							< 0.001
1 (least deprived)	9722 (21.77 %)	1963 (21.98 %)	2049 (22.94 %)	2042 (22.86 %)	1924 (21.54 %)	1744 (19.53 %)	
2	9432 (21.12 %)	1955 (21.89 %)	1893 (21.20 %)	1947 (21.80 %)	1910 (21.39 %)	1727 (19.34 %)	
3	8928 (19.99 %)	1812 (20.29 %)	1807 (20.23 %)	1738 (19.46 %)	1774 (19.86 %)	1797 (20.12 %)	
4	8624 (19.31 %)	1657 (18.55 %)	1721 (19.27 %)	1692 (18.95 %)	1736 (19.44 %)	1818 (20.36 %)	
5 (most deprived)	7949 (17.80 %)	1544 (17.29 %)	1461 (16.36 %)	1512 (16.93 %)	1587 (17.77 %)	1845 (20.66 %)	
Education, n(%)							< 0.001
Higher degree	22,798 (51.05 %)	4871 (54.54 %)	4833 (54.11 %)	4680 (52.40 %)	4515 (50.55 %)	3899 (43.66 %)	
Any school degree	12,098 (27.09 %)	2221 (24.87 %)	2285 (25.59 %)	2370 (26.54 %)	2522 (28.24 %)	2700 (30.23 %)	
Vocational qualification	7044 (15.77 %)	1287 (14.41 %)	1289 (14.43 %)	1394 (15.61 %)	1371 (15.35 %)	1703 (19.07 %)	
None of the above	2715 (6.08 %)	552 (6.18 %)	524 (5.87 %)	487 (5.45 %)	523 (5.86 %)	629 (7.04 %)	
Smoking status, n(%)							< 0.001
Never	24,052 (53.86 %)	5027 (56.29 %)	5018 (56.19 %)	4825 (54.03 %)	4704 (52.67 %)	4478 (50.14 %)	
Previous	17,071 (38.23 %)	3515 (39.36 %)	3417 (38.26 %)	3480 (38.97 %)	3475 (38.91 %)	3184 (35.65 %)	
Current	3532 (7.91 %)	389 (4.36 %)	496 (5.55 %)	626 (7.01 %)	752 (8.42 %)	1269 (14.21 %)	
Physical activity, n(%)							< 0.001
Low	8840 (19.80 %)	1229 (13.76 %)	1600 (17.92 %)	1846 (20.67 %)	2022 (22.64 %)	2143 (24.00 %)	
Moderate	23,520 (52.67 %)	4743 (53.11 %)	4908 (54.95 %)	4738 (53.05 %)	4715 (52.79 %)	4416 (49.45 %)	
High	12,295 (27.53 %)	2959 (33.13 %)	2423 (27.13 %)	2347 (26.28 %)	2194 (24.57 %)	2372 (26.56 %)	
Cancer history (yes), n(%)	1267 (2.84 %)	249 (2.79 %)	256 (2.87 %)	268 (3.00 %)	270 (3.02 %)	224 (2.51 %)	0.23

DP – Dietary Pattern; FM – Fat Mass; aSMM – Appendicular Skeletal Muscle Mass; BMI – Body Mass Index; WC – Waist Circumference; WHR – Waist-to-Hip Ratio; *Pearson’s chi-squared tests were used to compare the distribution between z-score for categorical variables, and ANOVA was used to compare the z-score for continuous variables.

### Associations between DP and baseline body composition and adiposity

**Fig. 2:**
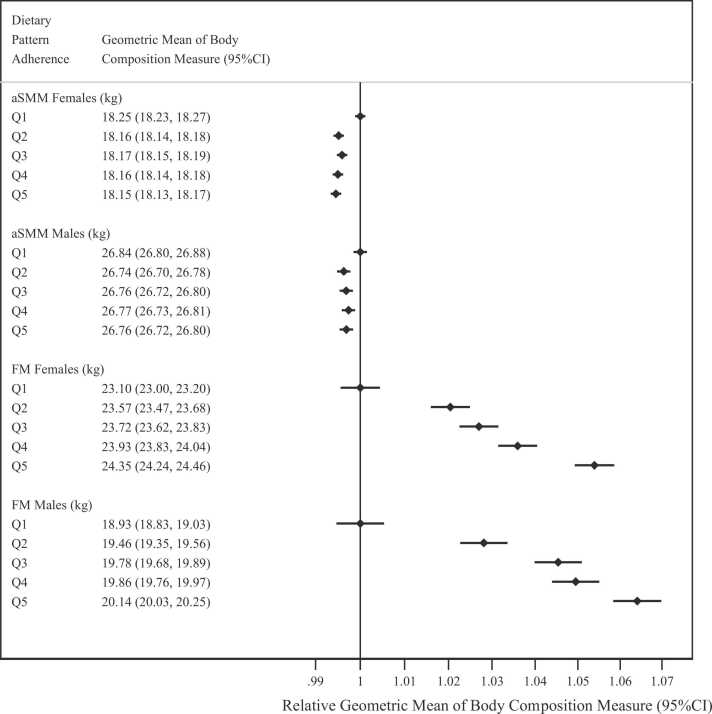
**Geometric mean estimates of appendicular skeletal muscle mass (kg) and fat mass (kg) measured at baseline by dietary pattern quintile.** * Q1–Q5 refer to the dietary pattern quintiles; FM – Fat Mass; aSMM – Appendicular Skeletal Muscle Mass; P_sex-interaction_ aSMM < 0.0001; P_sex-interaction_ FM < 0.0001.

There were significant positive associations between DP quintile and geometric mean estimates of FM, BMI and WC measured at baseline (Fig. 2 and Supplementary Figs. 2–4). There was a significant inverse association between DP quintile and aSMM in both sexes, and a significant positive association between DP adherence and WHR, although the differences in mean estimates across quintiles were minimal (Supplementary Fig. 4).

### DP adherence and changes in body composition and adiposity

**Fig. 3:**
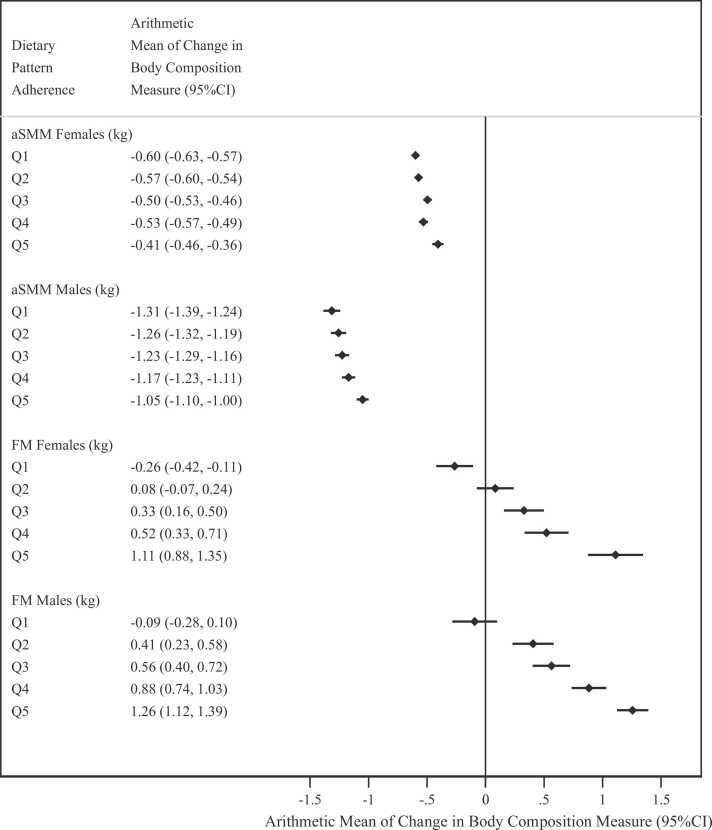
**Arithmetic mean estimates of the change between baseline and follow up in appendicular skeletal muscle mass (kg) and fat mass (kg) by dietary pattern quintile.** * Q1–Q5 refer to the dietary pattern quintiles; FM – Fat Mass; aSMM – Appendicular Skeletal Muscle Mass; P_sex-interaction_ aSMM < 0.0001; P_sex-interaction_ FM = 0.001.

The prospective analysis quantified the average change between the first and last follow-up in body composition and fat distribution measurements (8.1 years for both sexes, with a minimum of 2.2 and maximum of 13.8 years) in the subsample of people with follow up measurements (Fig. 3). In both sexes, there were significant positive associations between DP quintile and changes in FM (*P < 0.001)*. The predicted mean change in FM amongst women in Q1 of the DP was − 0.26 kg (− 0.42 to − 0.11), vs 1.11 kg (0.88–1.35) in Q5, while for men the change in Q1 was − 0.09 kg (− 0.28 to 0.10) vs 1.26 kg (1.12–1.39) in Q5 of the DP (Fig. 3). The associations between DP adherence and changes in aSMM were significantly positive albeit the changes across quintiles were very small (Fig. 3).

**Fig. 4:**
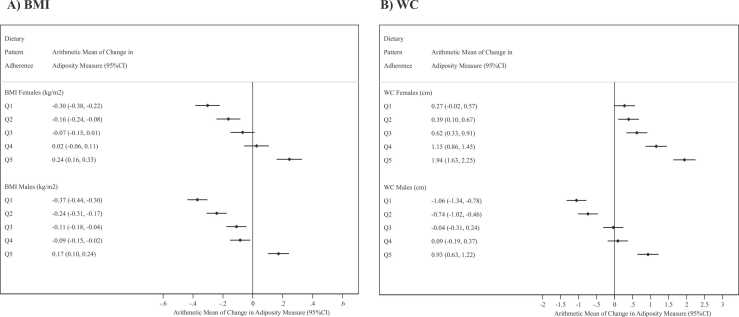
**Arithmetic mean estimates of the change between baseline and follow up in body mass index (kg/m**^**2**^**) and waist circumference by dietary pattern quintile.** * Q1–Q5 refer to the dietary pattern quintiles; BMI – Body Mass Index; WC – Waist Circumference; P_sex-interaction_ BMI = 0.097; P_sex-interaction_ WC = 0.252.

Similarly there were significant positive associations between DP quintile and changes in BMI and WC in both sexes (*P* < 0.001) (Fig. 4). The predicted mean change in BMI amongst women in Q1 was − 0.30 kg/m^2^ (− 0.38 to − 0.22), compared to 0.24 (0.16–0.33) in Q5. The corresponding predicted mean change in BMI for men in Q1 was − 0.37 (− 0.44 to 0.30) vs 0.17 (0.10–0.24) in Q5 (Fig. 4**A**). For WC, women in Q1 changed an average of 0.27 cm (− 0.02 to 0.52) over the follow-up period, whilst those in Q5 gained an average of 1.94 cm (1.63–2.25); and men in Q1 lost an average of − 1.06 cm (− 1.34 to − 0.78) whilst those in Q5 gained an average of 0.93 cm (0.63–1.22) over the follow-up period (Fig. 4**B**). There was a positive association between DP quintile and change in WHR in both sexes although the differences in mean estimates across quintiles were small (Supplementary Fig. 1).

Mean estimates from the sensitivity analysis (excluding those with a BMI ≥ 40 kg/m^2^, those who had not completed a 24-h dietary assessment at baseline, and those with fewer than 3 WebQ’s) were consistent with the results from the main analysis (Supplementary Tables 2–7).

## Discussion

In this sample of British adults, a higher adherence to an unhealthy DP (characterised by high intakes of chocolate confectionery, butter and refined carbohydrates, and low intakes of fruits and vegetables), was associated with significant increases in FM, WC and BMI in both men and women over an average of 8.1 years of follow up. Consistent associations were observed with baseline measurements of FM, BMI, and WC in both men and women.

Previously identified unhealthy dietary patterns, which are similar to the DP being studied in the current study and include those named as “Western”, “Modern” or “Unhealthy” dietary patterns, were also characterised by high intakes of food groups such as sugary food products, savoury snacks, margarine, or high-fat cheese (Supplementary Table 1). None of these previous longitudinal studies have investigated BIA-derived body composition measures (FM and aSMM) as outcomes. Both men and women adhering the most to the DP in this study had higher estimated gains in FM than those in adhering the least, with females in Q1 having estimated losses of FM, despite the natural gain in FM observed with increasing age [23]. This DP is fundamentally characterised by a higher consumption of energy-dense foods, saturated fatty acids and sugars [2], all of which contribute to higher average energy intake, which likely explain the higher body weight, 60–80 % of which is attributable to an increase in FM [24], [25]. Higher consumption of free sugars can also promote hyperinsulinemia, promoting the uptake of glucose and fatty acids into the adipose tissue, and increasing adiposity [26].

Conversely, fibre consumption was inversely related to the DP quintiles. Fibre promotes satiety without increasing the energy content of a meal, thus preventing excess consumption which leads to weight gain and obesity [27]. On the other hand, the lack of clinically important associations (prospective or cross-sectional) between this DP and aSMM is plausible and could be related to the nature of this DP which did not show very high or low factor loadings for protein-rich foods.

In terms of other classical adiposity measures, our results are consistent with a small longitudinal study in Chinese adults examining the association between dietary patterns derived by principal component analyses and adiposity outcomes (n = 1085), which found that those in the highest quartile of a “Modern” DP had a 0.29 kg/m^2^ increase in BMI, and 1.44 cm increase in WC over the 7-year follow-up period [4]. A recent study in the UK Biobank population derived DPs characterised by fat type, and showed that a DP high in SFA but low in MUFA/PUFA foods (butter and high fat cheese) was significantly associated with the incidence of obesity and abdominal obesity (WC men: ≥ 102 cm; women: ≥ 88 cm) after 6 years of follow up, which is consistent with our results given that our DP shared similar food groups than this other DP characterised by fat type [28].

Cross-sectional associations between “Western”, “Modern” or “Unhealthy” dietary patterns and aSMM have also showed both inverse and positive associations. Our associations with baseline measurements are consistent with a 2019 cross-sectional study in Korean adults (n = 3488), which found no association between adherence to a Western Diet and aSMM in males or females [29]. However, a study in American males (n = 903), found that a higher adherence to a Western diet was associated with a higher total body muscle mass, possibly because this study did not adjust for the confounding effect of FM, or because of the small sample size, meaning random error is more likely [30]. We found a positive cross-sectional association between FM and DP quintile, which is consistent with most of the existing literature [31], [32]. Furthermore, several cross-sectional studies have generally found significant positive associations between adherence to a Western/Modern/Unhealthy dietary pattern and measures of adiposity (BMI, WC and WHR) [2], [32], [33]. The cross-sectional and prospective associations between DP quintile and BMI were similar to, but slightly weaker than, the association found with FM. This is expected given that BMI includes both FM and aSMM, and since the latter shows no association with DP quintile, this may be attenuating the overall strength of the association between DP quintiles and BMI.

In previous studies which have stratified by sex, there were differences in some [5], [34], but not all [33], associations for men and women. In this study, we found evidence for effect modification by sex in most of the associations, with women having a greater increase in FM, BMI and WC as DP quintile increased compared to men in the cross-sectional association, but men having a greater increase in the change in FM as DP quintile increases. Some effect modification by sex is plausible, given that there are well-evidenced sex differences in body composition, such as the higher proportion of body FM in women compared to men, which affect energy metabolism [35].

The major strength of this study is the use of a large contemporary cohort of British adults with detailed dietary data as well as body composition measurements. Of all the previous studies on dietary patterns and body composition, this is the first to report associations with DPs derived through reduced rank regression (RRR). Compared to other exploratory DP approaches, RRR is particularly useful to include a priori knowledge of nutrient-disease associations to derive data-driven DPs that are associated with disease endpoints [7], [36].

In terms of limitations, as with all observational studies, some degree of self-selection, or healthy volunteer bias may be present. The self-reported dietary measurements are prone to recall bias and misreporting, however, by only including participants who had completed a minimum of two WebQ’s, we ensured that dietary data better reflected usual intake than a single measurement would have [12], [13], [37]. Our sensitivity analysis on the sample of people providing three or more 24-h dietary questionnaires showed similar results. Dietary data was collected over a 2-year period, but the prospective analysis had an average of 8.1 years of follow-up, potentially leading to measurement error if people changed their diets after the collection of dietary data. Some of the confounders (including height) were measured at the baseline assessment by trained interviewers, reducing the measurement error which may occur if these were self-reported. In any case, inaccurate measurement of these covariates or residual confounding cannot be ruled out. Although we reported a large variability in the number of years between baseline and follow up measurements, follow-up time was not related to the DP quintiles. Finally, the cross-sectional analyses are susceptible to reverse causality, but the prospective analyses help support the observed cross-sectional associations. However, the prospective analyses relied on a smaller number of people with available data at follow up, which may have limited the representativeness of this cohort.

In conclusion, this study provides evidence that higher adherence to an unhealthy DP previously identified among middle-aged British adults, is positively associated with significant gains in fat mass, overall adiposity and waist circumference, over and above the natural changes in body composition observed with age [23]. These changes in body composition among those adhering more to this DP help explain the previously observed associations with CVD, diabetes and all-cause mortality.

## CRediT authorship contribution statement

**AS, APC, JC and CP** Conceptualization; **AS, MG and CP** Data curation; **AS and CP** Formal analysis; **CP** Funding acquisition; **AS, APC, JC and CP** Investigation; AS, APC, JC, MG, SJ and CP Methodology; **AS, APC, JC and CP** Supervision; **AS, APC, JC and CP** Writing – original draft; **AS, APC, JC, MG, SJ and CP** Writing – review & editing.

All authors revised it critically for intellectual content and approved the final draft. The corresponding author attests that all listed authors meet authorship criteria and that no others meeting the criteria have been omitted. All authors warrant that the article is the authors' original work, hasn't received prior publication and isn't under consideration for publication elsewhere.

## Ethics approval and consent to participate

The UK Biobank study was conducted according to the Declaration of Helsinki and ethical approval was granted by the North West Multi-Centre Research Ethics Committee (Reference no. 06/MRE08/65). At recruitment, all participants gave informed consent to participate and be followed-up through data-linkage.

## Funding

CP received a British Nutrition Foundation Drummond 2016 pump priming award which paid for access to the data. CP is currently funded by a Ramon y Cajal Fellowship RYC2020-028818-I (Ministry of Science and Innovation, Spain). SAJ is a NIHR Senior Investigators and funded by the Oxford NIHR Biomedical Research Centre; MG and SAJ are funded by the NIHR Oxford and Thames Valley Applied Research Collaboration and NIHR Biomedical Research Centre. APC is supported by a Cancer Research UK Population Research Fellowship (C60192/A28516) and by the World Cancer Research Fund (WCRF UK), as part of the Word Cancer Research Fund International Grant Programme (2019/1953). JC is supported by core grants to CTSU (Clinical Trial Service Unit) from the Medical Research Council and the British Heart Foundation (CH/1996001/9454). UK Biobank was established by the Wellcome Trust, Medical Research Council, Department of Health, Scottish government, and Northwest Regional Development Agency. It has also had funding from the Welsh assembly government and the British Heart Foundation. The funders had no role in designing the study, the analysis, or the decision to submit the paper. The views expressed are those of the authors and not necessarily those of the NIHR or the Department of Health and Social Care.

## Data Availability

UK Biobank data is available to researchers on application (https://www.ukbiobank.ac.uk/enable-your-research).
