# Supplementary material for: Dietary pattern adherence in association with changes in body composition and adiposity measurements in the UK Biobank study
Source: Obes Res Clin Pract. 2023 May-Jun;17(3):233–41. doi: 10.1016/j.orcp.2023.05.008 (PMC10659985; doi:10.1016/j.orcp.2023.05.008)
Supplement: Supplementary file 1 — Supplementary material. [file mmc1.docx]

**Supplementary Table 1**

**Supplementary Table 1 cont.**

**Supplementary Table 1 cont.**

**Supplementary Table 1 cont.**

**Supplementary Table 1 cont.**

**Supplementary Table 1 cont.**

**Supplementary Table 1 cont.**

**Supplementary Figure 1. Mean estimates of the change in WHR by DP quintile**

* Q1 to Q5 refer to the dietary pattern quintiles, WHR – Waist to Hip Ratio; P_sex-interaction_ WHR =0.443

**Supplementary Figure 2. Mean estimates of BMI (kg/m2) from cross-sectional multivariable linear regressions, by DP quintile.**

* Q1 to Q5 refer to the dietary pattern quintiles; BMI – Body mass index; P_sex-interaction_ BMI <0.0001

**Supplementary Figure 3. Mean estimates of WC (cm) from cross-sectional multivariable linear regressions, by DP quintile.**

* Q1 to Q5 refer to the dietary pattern quintiles; WC – Waist Circumference; P_sex-interaction_ WC =0.0001

**Supplementary Figure 4. Mean estimates of WHR from cross-sectional multivariable linear regressions, by DP quintile.**

* Q1 to Q5 refer to the dietary pattern quintiles; WHR – Waist to Hip Ratio; P_sex-interaction_ WHR =0.0004

**Supplementary Table 2. Mean estimates from fully-adjusted multivariable linear regression of aSMM (kg) and FM (kg), by DP quintile, and stratified by sex, excluding those with BMI>40 kg/m^2^**

*Fat mass [FM], appendicular skeletal muscle mass [aSMM]

**Supplementary Table 3. Mean estimates from fully-adjusted multivariable linear regression of aSMM (kg) and FM (kg), by DP quintile, and stratified by sex, excluding those who did not have their first WebQ at baseline.**

*Fat mass [FM], appendicular skeletal muscle mass [aSMM]

**Supplementary Table 4. Mean estimates from fully-adjusted multivariable linear regression of aSMM (kg), FM (kg), by DP quintile, and stratified by sex, excluding those who do not have at least 3 WebQ’s.**

*Fat mass [FM], appendicular skeletal muscle mass [aSMM]

**Supplementary Table 5. Mean estimates from fully-adjusted multivariable linear regression of BMI (kg/m^2^), WC (cm) and WHR, by DP quintile, and stratified by sex, excluding those with a BMI > 40 kg/m^2^.**

*Body mass index [BMI], waist circumference [WC], waist-to-hip ratio [WHR]

**Supplementary Table 6. Mean estimates from fully-adjusted multivariable linear regression of BMI (kg/m^2^), WC (cm) and WHR, by DP quintile, and stratified by sex, excluding those who do not have at least 3 WebQ’s.**

*Body mass index [BMI], waist circumference [WC], waist-to-hip ratio [WHR]

**Supplementary Table 7. Mean estimates from fully-adjusted cross-sectional multivariable linear regression of changes in BMI (kg/m^2^) WC (cm) and WHR, by DP quintile, and stratified by sex, excluding those who do not have a WebQ’s at baseline.**

*Body mass index [BMI], waist circumference [WC], waist-to-hip ratio [WHR]
